# Refractory axillary venous spasm during permanent pacemaker implantation

**DOI:** 10.1186/s43044-020-00102-z

**Published:** 2020-10-20

**Authors:** Krishna Santosh Vemuri, Nitin Parashar, Dinakar Bootla, Pruthvi C. Revaiah, Kewal Kanabar, Krishna Prasad Nevali, Yash Paul Sharma, Ganesh Kasinadhuni, Prashant Panda

**Affiliations:** 1 Department of Cardiology, Postgraduate Institute of Medical Education and Research, Chandigarh, Chandigarh, India; 2 Department of Cardiology, All India Institute of Medical Sciences, New Delhi, New Delhi, India

**Keywords:** Complete heart block, Axillary venous spasm, Refractory venous spasm, Case report

## Abstract

**Background:**

Vascular spasm is well known to occur in the arterial system. Central venous spasm during pacemaker implantation is uncommon with only a few cases reported from time to time. Sometimes, the venous spasms may not respond to nitroglycerine injections which requires a change of access site and undue discomfort for the patient.

**Case presentation:**

A 72-year-old female patient with no prior comorbidities presented to us with recurrent dizziness on exertion and at rest. The electrocardiogram showed complete heart block, likely to be of sclerodegenerative etiology as the patient did not have any ischemic symptoms, also the electrocardiogram and echocardiogram did not show any evidence of ischemia. As part of the hospital protocol, a venogram was performed by giving intravenous diluted contrast (iohexol) through the left brachial vein, which showed good-sized axillary and subclavian veins. We attempted to cannulate the left axillary vein with a 16G needle using Seldinger technique, but the axillary vein could not be cannulated despite multiple attempts. We gave incremental boluses of intravenous nitroglycerine, despite that the left axillary vein could not be cannulated. Repeat intravenous contrast injection showed severe spasm of axillary and subclavian veins. Finally, the axillary vein was cannulated from the right side using anatomical landmarks and a pacemaker was implanted.

**Conclusions:**

Venous spasm during device implantation although uncommon, it should be anticipated in patients with difficult cannulation to prevent inadvertent complications like pneumothorax and arterial injuries. Mild venous spasm may relieve with time but severe venous spasm may require a change of access site

## Background

Vascular spasm is commonly known to occur in the arterial system. Central venous spasm during pacemaker implantation is quite uncommon, with only a few cases reported previously in the literature. Moreover, venous spasms might not respond to nitroglycerin which is commonly used as a successful therapeutic agent for arterial spasms. Venous spasm may be related to the chemical effect of a contrast agent or the mechanical effect of multiple punctures and guidewire placement.

### Case presentation

A 72-year-old female patient having no prior comorbidities presented to us with recurrent episodes of dizziness both on exertion as well as at rest. The electrocardiogram showed a complete heart block with narrow QRS complexes (QRS duration 110 ms) and an escape rate of 40 beats per minute. A presumptive diagnosis of “sclero-degenerative complete heart block” was made. There was no history suggestive of acute coronary syndrome or stable coronary heart disease. Electrocardiogram did not reveal significant ST–T wave changes and the echocardiogram showed normal left ventricular function without structural heart disease. Her serum electrolytes and renal function parameters were within the normal range.

**Fig. 1:**
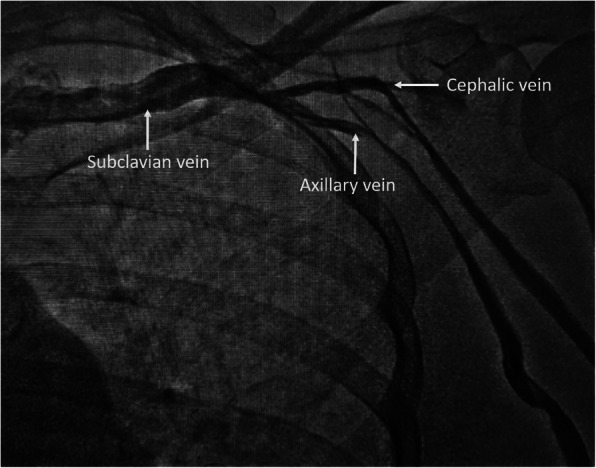
Intravenous contrast injection from the left brachial vein showing good-sized axillary and subclavian veins

**Fig. 2:**
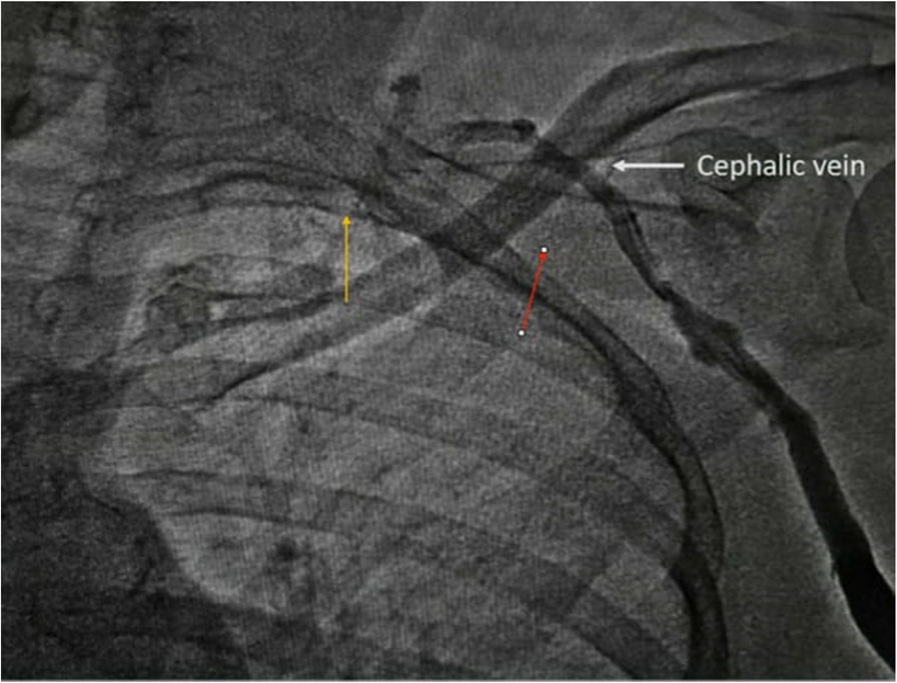
Second intravenous contrast injection from the left brachial vein showing severe axillary and subclavian venous spasm (axillary and subclavian veins almost disappeared) with compensatory dilatation of cephalic vein due to increased venous flow

**Fig. 3:**
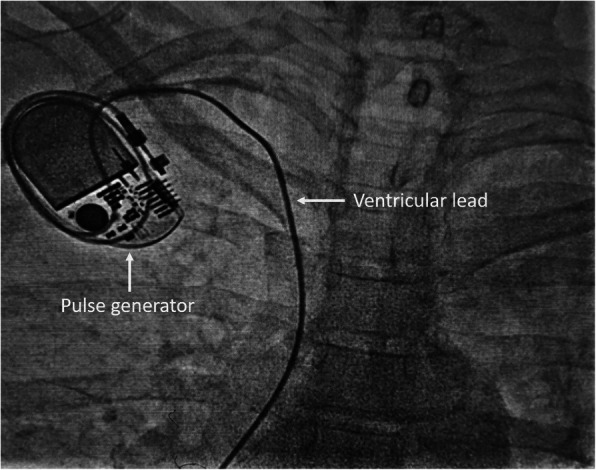
Final implantation of single-chamber permanent pacemaker from the right side

A temporary pacemaker was inserted through the right femoral venous route and she was subsequently planned for permanent single-chamber pacemaker implantation. A Dual-chamber pacemaker, although ideal, was refused by the patient due to financial reasons. As part of the hospital protocol, an intravenous contrast (iohexol; 5 ml diluted in 5 ml normal saline) injection was given through the left brachial vein to delineate the venous anatomy and drainage of left upper limb veins, which showed good-sized axillary and subclavian veins draining into the left brachiocephalic vein and then into superior vena cava (Fig. 1). We made the skin incision in the left infraclavicular fossa and prepared the subcutaneous pocket for device placement. Then, we proceeded to an axillary venous puncture with 16 G needle using the Seldinger technique. However, the axillary vein could not be cannulated despite multiple attempts. To find out the cause, we gave another contrast injection in the left brachial vein which revealed severe spasm of axillary and subclavian veins (Fig. 2). Then, axillary vein puncture was attempted after 15 min of giving two sequential boluses of intravenous nitroglycerin (200 mcg followed by 400 mcg) with a gap of 5 min, still, the axillary vein could not be cannulated. A venogram was not done on the right side to avoid the risk of venous spasm and the axillary vein was cannulated using anatomical landmarks, and the pacemaker was successfully implanted in the right infraclavicular pocket (Fig. 3). Active fixation lead was screwed in right ventricular septum and the device was placed in a right infraclavicular pocket; the VVIR mode for pacing was selected. Skin incisions on both sides of the chest were closed in layers.

After this, the post-procedural period was uneventful. Pacemaker interrogation revealed normal pacing parameters. The next day, the ultrasonogram of the left upper limb was performed which revealed normal flow through the left axillary and subclavian veins without any evidence of local hematoma. Sutures were removed on the tenth day of the procedure. One week later, the patient was followed up in the outpatient department. There was no swelling at the local site or in any of the upper limbs. The patient remained well on follow-up after 3 months.

## Discussion

Peripheral venous spasm has been well documented during cardiac catheterization procedures with an incidence of 2%, usually occurring in small and tortuous vein s[1]. It has also been reported to occur during peripheral venous access too. Saphenous venous graft spasm causing recurrent angina has also been reporte d[2, 3]. Central venous spasm during pacemaker implantation is an uncommonly reported event, with very few case reports and a prospective study reported in the literatur e[4–7]. The incidence of venous spasm may vary from 5 to 8% depending on definitio n[6, 7]. In unfortunate cases of severe venous spasm, many times, the change of access site is required for pacemaker implantation which is associated with increased risk of complications and additional morbidity to the patien t[5, 8].

Differential diagnoses in our case include (1) venous thrombosis, which is unlikely as the patient was on anticoagulation for an implanted temporary pacemaker, (2) local hematoma causing compression of the axillary and subclavian veins but this is also less likely as second contrast injection showed absent contrast flow starting from the distal axillary vein, (3) severe venous spasm secondary to chemical effect of contrast agent is the most plausible condition in this case as entire course axillary and subclavian veins was not seen on second contrast injection. Venous spasm may also occur due to the mechanical effect of multiple punctures which is also unlikely in this patient.

The exact mechanism of venous spasm is not clearly understood but it may be related to the chemical effect of the contrast, mechanical effect of multiple needle punctures, and guidewire placement; the role of the sympathetic nervous system has also been considered. Some authors have suggested activation of platelets and mast cells in saphenous venous grafts leading to spasm. In a prospective study by Xuan et al., the severe venous spasm was found to be more common in female patients, age ≥70 years, and with an initial right-side punctur e[6]. Anatomically axillary and subclavian veins are deep-seated medium-sized veins, with its tunica media containing circularly arranged smooth muscle fibers that mediate vascular contraction or relaxation. The nature and extent of venous spasm may also be regulated by local autonomic and metabolic factor s[9].

There are no well-proven preventive or immediate treatment measures for venous spasm. Multiple strategies including intravenous nitroglycerine, calcium channel blocker, procaine, and sedatives like diazepam might have some preventive effect or shorten the spasm duratio n[7, 8, 10]. The efficacy of nitroglycerin for treating and preventing arterial spasm has been well established,[11, 12] but its use for the treatment of venous spasm is controversial. Xuan et al. found that pre-treatment with intravenous nitroglycerin was an effective way for the prevention of venous spasm during contrast-guided axillary venous punctur e[8]. In most cases, a partial relief of spasm occurs over time, hence careful venous puncture can be attempted again after some time. However, a severe spasm, as in our case, may considerably alter the course of the procedure.

## Conclusions

- Venous spasm during device implantation is an uncommon phenomenon but should be anticipated in patients with difficult cannulation of access vein.
- Mild venous spasm may relieve spontaneously with time or with the use of nitroglycerin, but a severe venous spasm alters the course of the procedure, and different access sites might be required.
- Understanding and anticipation of this complication might prevent inadvertent local injuries and other life-threatening complications like pneumothorax, arterial punctures causing bleeding or hematoma.

## Data Availability

Not applicable.
